# Sex/Gender Differences in Camouflaging in Children and Adolescents with Autism

**DOI:** 10.1007/s10803-020-04615-z

**Published:** 2020-07-20

**Authors:** Henry Wood-Downie, Bonnie Wong, Hanna Kovshoff, William Mandy, Laura Hull, Julie A. Hadwin

**Affiliations:** 1 Centre for Innovation in Mental Health – Developmental Lab, School of Psychology, University of Southampton, Highfield, Southampton, S017 1BJ UK; 2 Research Department of Clinical, Educational & Health Psychology, University College London, London, UK; 3 Present Address: Centre for Research in Inclusion, Southampton Education School, University of Southampton, Building 32, Southampton, SO17 1BJ UK; 4 West Sussex Educational Psychology Service, West Sussex County Council, 3rd Floor County Hall North, Chart Way, Horsham, RH12 1XH UK; 5 Present Address: East Sussex Educational Psychology Service, East Sussex County Council, Ocean House, 87-89 London Road, St Leonards-On-Sea, TN37 6DH UK; 6 Present Address: School of Education, Eden Building, Liverpool Hope University, Liverpool, L16 9JD UK

**Keywords:** Camouflaging, Compensation, Masking, Autism, Sex/gender differences, Reciprocity, Theory of mind

## Abstract

This study investigated sex/gender differences in camouflaging with children and adolescents (*N* = 84) with and without an autism diagnosis/increased levels of autistic traits using two conceptualisations/operationalisations of camouflaging. A significant group-by-gender interaction using ANCOVA, with the covariate of verbal IQ, reflected similar levels of social reciprocity in autistic and neurotypical females, whereas autistic males had lower reciprocity than neurotypical males. Autistic females also had higher reciprocity than autistic males, despite similar levels of autistic traits (behavioural camouflaging). Additionally, autistic males and females had similar theory of mind skills, despite females having increased reciprocity (compensatory camouflaging). These findings provide evidence of increased camouflaging in autistic females, which may contribute to delay in the recognition of difficulties and provision of support.

Autism is a neurodevelopmental condition characterised by difficulties with social interaction and communication, as well as restricted and repetitive patterns of behaviour, activities and interests (American Psychiatric Association 2013). Autism is diagnosed in approximately 1% of the population and this diagnosis is made more frequently and earlier in development in males than females, with a reported approximate ratio of four males to every female (Fombonne 2009). Relatively recent large-scale population studies have, however, reported a ratio of approximately three males to every female (e.g., Baxter et al. 2015; Loomes et al. 2017; Zablotsky et al. 2015). This shift in recognition of autism in females fits with a growing body of research which reflects an increased clinical awareness of the female autism phenotype (Lai et al. 2016). This research suggests that autism may manifest differently between sexes/genders^1^, and has led some researchers to suggest there may be a female-specific phenotype of autism (e.g., Lai et al. 2015). Research studies have increasingly aimed to explore the possibility that there may be a differential phenotypic profile in autism between males and females (e.g., Hull et al. 2019a; Lai et al. 2016; van Ommeren et al. 2017).

Camouflaging, defined as strategies used to appear less autistic in social interactions (Hull et al. 2017), is argued to be a key feature of the female autistic^2^ phenotype (Hull et al. 2019a; reviews by Hull et al. 2020; Wood and Wong 2017). This sex/gender difference may increase challenges to identify females with autism and contribute to the later diagnosis for this group (e.g., Begeer et al. 2013; Giarelli et al. 2010). Late diagnosis in general, and camouflaging in particular, have been associated with increased mental health difficulties (e.g., Hull et al. 2019b; Lai and Baron-Cohen 2015) and a risk marker for suicidality (Cassidy et al. 2018). It is therefore important to investigate sex/gender differences in camouflaging to facilitate earlier identification and planning of specialised support for females (Lai et al. 2016).

Several qualitative studies have explored the experiences of females diagnosed with autism and these have resulted in rich and detailed accounts of camouflaging (e.g., Bargiela et al. 2016; Cridland et al. 2014; Hull et al. 2017; Tierney et al. 2016). For example, Tierney et al. (2016) interviewed ten female adolescents with autism to explore their experiences of social relationships. All participants described using ‘masking’ strategies to appear more socially competent, which were often motivated by a desire for friendship. However, adolescents further indicated that the use of these cognitively demanding strategies resulted in adverse psychological consequences. For example, one adolescent described an ‘identity crisis’, attributed to ‘pretending to be the same as everyone else’ (Tierney et al. 2016, p. 79). In a further study, Cridland et al. (2014) interviewed three autistic female adolescents, their mothers, and two other mothers who also had autistic daughters. All the autistic females reported experiencing difficulties developing and maintaining friendships. In addition, maternal reports suggested that these challenges resulted, to some extent, from a reliance on imitation during childhood in an attempt to mask underlying social difficulties (Cridland et al. 2014).

Similar themes were found in a study that involved interviewing 14 autistic women diagnosed in late adolescence or adulthood (Bargiela et al. 2016). Detailed accounts of ‘pretending to be normal’ (p. 3287) were given in which young adults reported using explicit strategies to fit in with peers. These included using learnt phrases and facial expressions from TV, books and magazines, social imitation, and masking autistic traits. In addition, eight women indicated that when they were teenagers, their peers were noticeably more advanced in their social abilities, leading to difficulties forming friendships and feelings of rejection. Many women also reported having experienced a mental health condition, with depression, anxiety and eating disorders being the most common. Hull et al. (2017) interviewed 92 autistic adults of all genders about camouflaging, which often was motivated by a similar desire to fit in and connect with others. In addition, adults reported that camouflaging resulted in both negative (e.g. exhaustion, loss of identity) and, for a minority of participants, positive (e.g., connecting with others) consequences (Hull et al. 2017). In this research, camouflaging was reported in a similar number of males and females, as well as participants who identified as non-binary. However, it did highlight sex/gender differences in the techniques used for, and the consequences of, camouflaging.

## Measures of Social Camouflaging

Hull et al. (2019b) distinguished between two broad approaches to defining and measuring camouflaging, namely ‘discrepancy methods’ and ‘observational/reflective methods’. Discrepancy methods aim to measure the gap between external behavioural presentation (e.g., social skills) and internal measures of ability (e.g., theory of mind). On the other hand, observational/reflective methods involve measuring specific behaviours that constitute camouflaging, such as those that enable autistic individuals to blend into their social environment.

### Discrepancy Methods (‘Compensatory Camouflaging’)

We refer to discrepancy methods of measuring camouflaging as ‘compensatory camouflaging’; this is based upon Livingston and Happé’s (2017, p. 731) conceptualisation of compensation, defined as “the processes contributing to improved behavioural presentation of a neurodevelopmental disorder despite persisting core deficit(s) at cognitive and/or neurobiological levels”. Relatively few studies have investigated compensatory camouflaging (Livingston et al. 2018). Lai et al. (2016) found that the discrepancy between social behaviour (as measured by the Autism Diagnostic Observation Schedule (Lord et al. 2001) and self-reported autistic traits and theory of mind ability was significantly greater for adult autistic females than males. Specifically, theory of mind scores were similar between sexes/genders, but autistic females showed more advanced social communication skills. The paper provides evidence of higher levels of compensatory camouflaging in adult autistic females. Rynkiewicz et al. (2016) also found evidence for compensatory camouflaging in 5–10-year-olds, where girls (versus boys) with autism showed better non-verbal communication skills on two activities from the ADOS, despite having lower social-cognitive ability. Livingston et al. (2018) found that autistic (male and female) adolescents who demonstrated high levels of compensatory camouflaging (i.e., good social skills, despite poor theory of mind) had significantly higher IQ than those who did not show this differential profile. In addition, there were more females relative to males in the high (versus low) compensatory camouflaging group; the male-to-female ratio for individuals who showed little compensatory camouflaging was 4.71:1, compared with a ratio of 3.67:1 for individuals who demonstrated higher levels of compensatory camouflaging.

### Observational/Reflective Methods (‘Behavioural Camouflaging’)

We refer to observational/reflective methods of measuring camouflaging as ‘behavioural camouflaging’, as they involve measuring specific camouflaging behaviours. For example, Dean et al. (2017) examined the social behaviours of children, aged 7 years, both with and without autism on the playground. They found that autistic girls tended to stay near peers (without fully engaging with them), weaving in and out of activities. Similarly, neurotypical girls spent most of their time socialising with peers. In contrast, autistic boys spent most of their time alone, whilst neurotypical boys often played games together. Consequently, girls (and not boys) with autism appeared similarly to their neurotypical counterparts, providing evidence of greater levels of behavioural camouflaging in autistic girls. A further study found that autistic female (versus male) children and adolescents had significantly higher reciprocity scores—despite having similar levels of parent or teacher reported autistic symptoms—which was more similar to neurotypical children and adolescents (van Ommeren et al. 2017). Parish-Morris et al. (2017) investigated sex/gender differences in conversation fillers in autistic and neurotypical female and male children and adolescents. The results showed that autistic and neurotypical females displayed similar levels of vocalisations; specifically, they had similar “um ratios” (“um” usage relative to total amount of “um” and “uh”), while males with autism used this pragmatic marker significantly less than neurotypical males. This result was evident despite autistic male and female children and adolescents having comparable levels of parent-reported autistic traits. Collectively, reflective/observational methods have found that autistic females are more similar to neurotypical females, compared with autistic and neurotypical males, despite often having similar levels of autistic traits.

### Current Study

Research findings indicate that (compensatory and behavioural) camouflaging is greater in autistic females than males and, irrespective of sex/gender, higher levels of compensatory camouflaging are associated with increased IQ. However, studies that have explored the emergence of these behaviours in children and adolescents with autism are scarce. In addition, only one previous study has included children and adolescents with high levels of autistic traits who have not yet received a diagnosis (Livingston et al. 2018). We argue that individuals who are camouflaging are less likely to have received a formal diagnosis and especially in childhood, as they will have, to some extent, have masked their social difficulties. Deficits in social reciprocity are necessary to receive a diagnosis of Autism Spectrum Disorder (ASD) based on the Diagnostic and Statistical Manual of Mental Disorders, 5th Edition (APA, 2013) diagnostic criteria. Therefore higher levels of reciprocity observed in autistic females—as found by van Ommeren et al. (2017)—may be one way in which autistic females camouflage their social difficulties, thereby ‘flying under the radar’ (NASEN 2016). Accordingly, we used a measure of reciprocal interaction as an index of social behaviour and, in addition, a theory of mind measure to look at underlying social-cognitive ability.

This study aimed to replicate and extend existing research exploring camouflaging in children and adolescents with high levels of autistic traits, both with and without diagnoses of autism and with a focus on social reciprocity. We operationalised camouflaging using observational/reflective (behavioural camouflaging) and discrepancy (compensatory camouflaging) methods. We hypothesised that girls with autism would engage in higher levels of both behavioural and compensatory camouflaging. Specifically, we anticipated that any difference in reciprocity for girls with and without autism would be smaller than the difference between reciprocity scores for boys with and without autism, despite similar levels of autistic traits (i.e., behavioural camouflaging). In addition, we also anticipated that increased reciprocity in females (versus males) with autism would be evident, despite a similar level of social-cognitive ability (i.e., theory of mind) between sexes/genders (i.e., compensatory camouflaging). Finally, it was expected that IQ would be higher in children with autism who displayed higher (versus lower) levels of compensatory camouflaging, irrespective of sex/gender.

## Methods

### Power Analysis

The current study utilised the Interactive Drawing Task (IDT, van Ommeren et al. 2012) to provide an index of reciprocity. To our knowledge, only one published study has previously investigated sex/gender differences using this task (van Ommeren et al. 2017). A power analysis using G*Power (Faul et al. 2007) was conducted when designing the study, using the effect size of the difference between autistic and neurotypical males (*η*^*2*^_*P*_ = 0.24) from van Ommeren et al. (2017); this analysis showed that a minimum of 59 participants were needed to achieve 95% power.

### Participants

Special Educational Needs Coordinators (SENCos) and/or Head Teachers from 16 mainstream primary schools and three mainstream secondary schools in the South of England were approached to ask if their school would be interested in participating in the study. Of these, ten primary and two secondary schools agreed to participate. The reason cited for non-participation was lack of time (*n* = 4). SENCos from the participating schools either sent letters to parents of all eligible children and/or approached parents of children with an autism diagnosis. Parents were asked to read an information sheet, complete the Social and Communication Disorders Checklist (SCDC, Skuse et al. 2005; see measures below), and sign a consent form if they were happy for their child to take part. Children and adolescents also gave their written assent for participation.

Children who scored above the cut-off of 9 (as defined by Skuse et al. 2005; see measures below) on the SCDC were defined as having high autistic traits, and were included in the same group as those with an autism diagnosis (autism/high autistic traits group). These groups were created retrospectively after completion of the research. There were no significant differences between boys who had a clinical diagnosis and those who had high autistic traits (without a diagnosis) with respect to the two outcome measures of theory of mind (*t* = 1.26, *p* = 0.224, *d* = 0.54) or social reciprocity scores (*t* = 0.77, *p* = 0.450, *d* = 0.33). Similarly, there were no significant differences between girls who had a clinical diagnosis and those who had high autistic traits (without a diagnosis) with respect to theory of mind (*t* = 0.56, *p* = 0.582, *d* = 0.27) or social reciprocity scores (*t* = 0.60, *p* = 0.555, *d* = 0.28).

Table 1 shows the sample characteristics. The final sample comprised 84 children (22 boys with autism/high autistic traits, 18 girls with autism/high autistic traits, 22 neurotypical boys, 22 neurotypical girls) aged between 8–14 years. To test for pre-existing differences in verbal IQ, non-verbal IQ, full-scale IQ and age, we ran a series of 2 × 2 between subject ANOVAs comprising: 2 Sex/Gender (girls; boys) and 2 Group (neurotypical; autism/high autistic traits). Most main effects and interactions were non-significant (all *F*s < 2.52, all *p*s > 0.116), except for a main effect of group for verbal IQ (*F* = 6.64, *p* = 0.012), which indicated that neurotypical children and adolescents had significantly higher mean verbal IQ score than those with autism/high autistic traits.

**Table 1:** The mean (SD) and range and intercorrelations between reciprocal behaviour from the Interactive Drawing Test (IDT; van Ommeren et al. 2012, 2015) and social cognition from the Reading the Mind in the Eyes Test, Child’s Version (RMET-C; Baron-Cohen et al. 2001a, b) and IQ (full scale, verbal and non-verbal subtests) for male and female children and adolescents in the autism/high autistic traits and neurotypical groups

	Autism/high autistic traits (*n* = 40)				Neurotypical (*n* = 44)				All participants (n = 84)					
	Male (*n* = 22)		Female (*n* = 18)		Male (*n* = 22)		Female (*n* = 22)		Associations between variables (*r*)					
	Mean (SD)	Range	*M *(*SD*)	Range	*M *(*SD*)	Range	*M *(*SD*)	Range	1	2	3	4	5	6
1. Age (years)	10.08 (1.75)	8.08–13.92	10.12 (1.43)	7.92–13.42	10.50 (1.40)	8.58–14.42	9.62 (1.01)	8.08–11.5	1	.10	− .01	.19	.18	.17
*IQ*														
2. Full Scale IQ	99.55 (17.58)	76–142	99.00 (15.68)	71–120	107.59 (12.36)	84–128	101.41 (14.18)	70–119	.10	1	.88*	.89*	.08	.33**
3. Vocabulary	52.91 (10.03)	38–77	52.56 (12.16)	27–67	58.95 (7.15)	45–72	57.05 (7.77)	42–70	− .01	.88**	1	.57**	.05	.36**
4. Matrix reasoning	46.50 (12.34)	31–72	46.39 (9.16)	35–66	49.64 (8.27)	27–60	44.59 (10.05)	22–60	.19	.89**	.57**	1	.08	.22*
*Social behaviour and social-cognitive ability*														
5. Reciprocal behaviour	2.16 (1.13)	0.22–3.92	2.91 (0.99)	1.33–4.63	3.22 (1.05)	1.00–4.53	2.86 (1.12)	1.00–4.60	.18	.08	.05	.08	1	− .22*
6. Social cognition	17.68 (4.09)	9–26	17.56 (4.59)	8–24	19.05 (2.50)	15–23	18.32 (3.64)	12–26	.17	.33**	.36**	.22*	− .22*	1

*p < .05, **p < .01

Of the 22 boys in the autism/high autistic traits group, eight had a clinical diagnosis of ASD and two had a diagnosis of Asperger’s Syndrome, all confirmed by a paediatrician according to parental report. These boys scored highly on the parent-reported SCDC (*M* = 18.10, *SD* = 4.48) and SENCos confirmed they had seen copies of reports outlining their ASD diagnoses for all but one child. In addition, three boys were enrolled in a unit (within their mainstream school) that required them to have an autism diagnosis. The other 12 participants in this group exceeded cut-off on the SCDC (M = 14.33, SD = 3.99) and were either under assessment for ASD (*n* = 10), or concerns had been raised with respect to possible autism/social communication difficulties by school or parent (*n* = 2).

Of the 18 girls in the autism/high autistic traits group, eight had a clinical diagnosis of ASD confirmed by a paediatrician according to parental report. These girls scored highly on the SCDC (*M* = 17.43, *SD* = 5.19). The first author of this paper had seen a copy of the ASD diagnostic report for one girl, and SENCos confirmed they had seen copies of the reports outlining ASD diagnoses for the other seven girls. The other 10 participants in this group exceeded cut-off on the SCDC (*M* = 17.43, *SD* = 5.91) and were either under assessment (*n* = 8) or had been assessed for ASD and found to have a high level of traits just below clinical cut-off level (*n* = 1), or concerns had been raised with respect to possible autism/social communication difficulties by school and parent (*n* = 1).

### Measures

#### Autistic Traits

The SCDC (Skuse et al. 2005) is a 12-item parent-report screening checklist designed to measure autistic traits in the general population. Parents are asked to answer ‘not true’ (0), ‘quite or somewhat true’ (1), or ‘very or often true’ (2) to questions about their child’s behaviour in the last 6 months (e.g., ‘does not pick up on body language’). The possible score range is 0–24 and a score of nine or above suggests the individual may have autism. Skuse et al. (2005) found the SCDC to have excellent internal consistency (0.93), high test–retest reliability (0.83), good discriminative validity from other developmental disorders, and even better discrimination from non-clinical samples. In our sample, internal consistency, was acceptable for males with autism/high autistic traits (α = .72), neurotypical males (α = .74), and neurotypical females (α = .71). For females with autism/high autistic traits, internal consistency was good (α = .85).

#### Intelligence Quotient

We used the Wechsler Abbreviated Scale of Intelligence, Second Edition (WASI-II, Wechsler 2011) to measure intelligence. It includes verbal (Vocabulary) and non-verbal (Matrix Reasoning) subtests that collectively generate a full-scale IQ estimate. McCrimmon and Smith (2013) noted that the WASI-II has good-to-excellent internal consistency (0.87–0.91), acceptable to excellent test–retest stability (0.79–0.90), acceptable-to-excellent concurrent validity (0.71–0.92), excellent interrater reliability (0.94–0.99), as well as strong factor validity.

#### Social Reciprocity

We used the Interactive Drawing Test (IDT, van Ommeren et al. 2012, 2015) to measure social reciprocity. The IDT involves a real-life interaction in which the researcher and participant take turns to create a drawing. The IDT is as unstructured as possible in order to elicit spontaneous interaction, with the only one instruction being given (‘we are going to draw together’). The IDT generates a total score—based on the proportion of total number of turns—made up of the following four scales (Table 2 for further details): (1) reciprocal turn-taking (2) reciprocal interaction, (3) reciprocal interaction in the other’s initiative, and (4) reciprocal flexibility (for detailed information about the IDT see van Ommeren 2018). The maximum score for turn-taking is two, and is one for the other three scales; therefore total scores range from 0 to 5, with higher scores indicating higher levels of reciprocal behaviour. van Ommeren et al. (2015) found the IDT demonstrated excellent inter-rater reliability (0.95–1.00), moderate-to-good test retest reliability for the different subscales (0.47–0.70), and excellent criterion validity.

**Table 2:** Description of the four scales from the Interactive Drawing Test (IDT; van Ommeren et al. 2012, 2015) used to generate total social reciprocity score

Scale	Description
Reciprocal turn-taking	Children are awarded one point if they push the paper back, and two points if they push and rotate the paper back after they have finished their turn. In order to model reciprocal turn-taking behaviour, the researcher always pushes and rotates the paper back to the child after they have finished their turn
Reciprocal interaction	Children are awarded one point each time they contribute a meaningful element to a mutual object with the researcher. For example, both the researcher and child are contributing different elements to a house, such as windows and curtains
Reciprocal interaction in the other’s initiative	Children are awarded one point each time they contribute a meaningful element to an object initiated by the researcher. For example, the researcher first draws a tree, and the child then adds an apple to the tree
Reciprocal flexibility	Children are awarded points for accepting an (1) interfering, (2) absurd, and (3) destructive input, with a maximum of one point being awarded for accepting all three. For example, the absurd input involves the researcher adding two arms and a hand to the child or adolescent’s drawing. Child acceptance is defined as contributing to the researcher’s object, such as drawing the missing hand or colouring in the arms

To generate an index of overall reciprocal behaviour, the current paper focused on the total IDT score. The first and second authors of this paper were sent the administration guidelines by the first author of the test. They then practised administrating and scoring with adults and children, sending these to the first author of the IDT until she was confident that they had reached sufficient proficiency to use it with autistic children. In order to assess inter-rater reliability, 10 administrations of the IDT (five males with autism/high autistic traits; two females with autism/high autistic traits; two neurotypical males; one neurotypical female) were video recorded, and subsequently rated independently and blindly by the first and second authors. The intraclass correlation coefficient between these ratings was 0.94, indicating excellent reliability. For the final analysis, disagreements were resolved through discussion until a consensus was reached.

#### Social Cognition (Theory of Mind)

We used the Reading the Mind in the Eyes test, Child’s Version (RMET-C, Baron-Cohen et al. 2001a, b) as an index of social-cognitive ability to measure theory of mind (i.e., the ability to recognise the mental state of others). In this task, participants looked at 28 pictures of an individual when only their eye region was visible with four words written around it. Participants were read the four words and asked to choose the one ‘that best describes what the person in the picture is thinking or feeling’, with one answer being correct (score range = 0–28). The child’s version was developed from the adult version of the test (Baron-Cohen et al. 2001a, b), which has been used in over 250 published studies (Baker et al. 2014), including autism sex/gender difference research (e.g., Lai et al. 2016).

### Procedure

All children and adolescents took part in the study in a quiet room in their school and worked with one of two researchers. Before taking part, they were given an information sheet explaining the purpose of the study, which they were read if they were not proficient readers. Participants were reminded that they did not have to take part if they did not want to, that they could stop at any time, and that all information they gave was confidential. If children and adolescents were happy to take part, they were then asked to give their assent by signing a consent form.

All participants completed tasks in the following order: (1) IDT, (2) RMET-C, (3) WASI-II. In addition, all completed a further task (not reported in the current paper) that involved a semi-structured interview asking them about the drawing task they had completed, what a friend or family member would think of the drawing task, and to describe their favourite game or hobby. In total, the tasks took between 45 and 60 minutes to complete. All participants were then thanked, fully debriefed, and reminded they could still withdraw if they wished. No participants requested to withdraw from the study.

### Operationalisations of Camouflaging

We operationalised camouflaging using both an observational/reflective method (behavioural camouflaging) and discrepancy method (compensatory camouflaging). Specifically, camouflaging was operationalised as (1) autistic individuals appearing behaviourally similar to neurotypical peers, despite having underlying social difficulties (behavioural camouflaging) and (2) improved behavioural presentation despite underlying social-cognitive difficulties (compensatory camouflaging).

## Results

### Parametric Assumptions

One participant (neurotypical girl) had a very low total IDT score of 0.08 (2.49 standard deviations below the mean for neurotypical participants), and was investigated as a possible outlier. Scores in the neurotypical group (not including the potential outlier) ranged from 1.00–4.60 (*M* = 3.04, *SD* = 1.09), suggesting the outlier was from a different population, which would justify removal from the analysis (Field 2013). As a general rule of thumb, data points with a Cook’s distance of three times the mean are possible outliers (Algur and Biradar 2017); this participant had a Cook’s distance of 0.081, which is 6.75 times greater than the mean of 0.012, suggesting they were exerting undue influence on the overall results. Consequently, this participant was removed from analyses involving the IDT. For RMET-C scores, two possible outliers were investigated (both females with autism/high autistic traits); these participants were retained in the analysis as (1) there was no reason to believe they came from a different population than the one in question and (2) statistical results were the same whether they were included or excluded.

From inspection of histograms, three groups (males with autism/high autistic traits; females with autism/high autistic traits; neurotypical females) showed negative kurtosis for total IDT scores. However, all boxplots appeared symmetrical and Shapiro–Wilk tests were non-significant (all *D*s > 0.29, all *p*s > 0.089), suggesting the assumption of normality had been met. Levene’s test was non-significant (*F* = 0.88, *p* = 0.350), suggesting that the assumption of homogeneity of variance had been met.

For the RMET-C data, from inspection of histograms scores in the neurotypical male group showed signs of negative kurtosis, and females with autism/high autistic traits showed negative skew. However, all boxplots appeared symmetrical and Shapio-Wilk tests were non-significant for all groups (all *D*s > 0.93, all *p*s > 0.166) suggesting the assumption of normality had been met. Levene’s test was non-significant (*F* = 2.66, *p* = 0.107), suggesting the assumption of homogeneity of variance had been met.

### Observational/Reflective Method (‘Behavioural Camouflaging’)

**Fig. 1:**
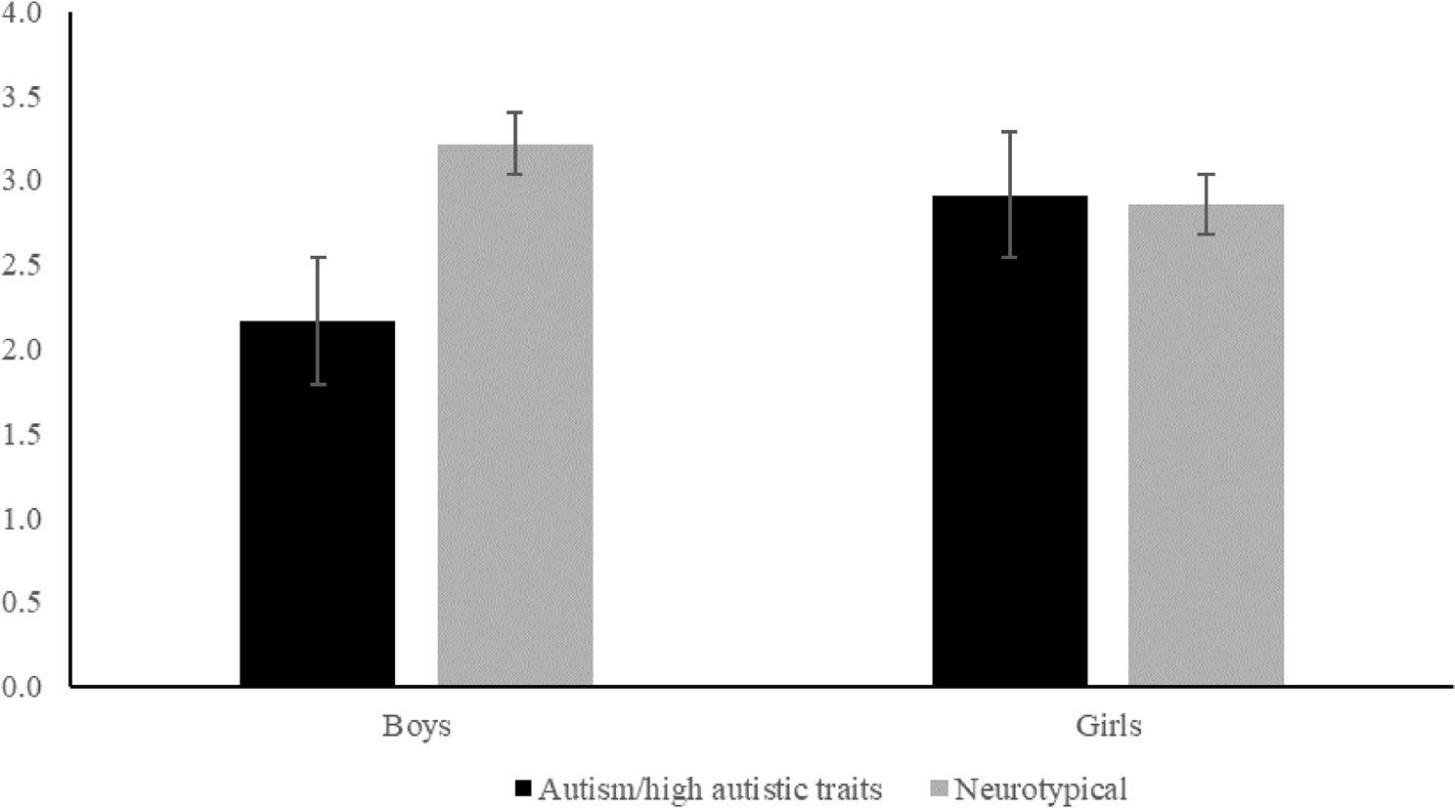
Total reciprocity scores (Interactive Drawing Test (IDT); van Ommeren et al. 2012, 2015) and standard errors for male and female children and adolescents in the autism/high autistic traits and neurotypical groups

The mean, SD and score range for the IDT task is shown in Table 1. As verbal IQ differed between the neurotypical and autism/high autistic traits groups, verbal IQ was entered as a covariate in this analysis. A 2X2 between-subjects ANCOVA was conducted for total IDT scores for 2 Sex/Gender (female, male) and 2 Group (neurotypical; autism/ high autistic traits), with the covariate of verbal IQ. This analysis showed a significant main effect of group (*F*(1, 78) = 4.29, *p* = 0.042, *η*^*2*^_*P*_ = 0.052), reflecting lower total IDT scores in the autistic/high autistic traits, compared to neurotypical group (see Fig. 1). There was no significant main effect of sex/gender (*F*(1, 78) = 0.65, *p* = 0.425, *η*^*2*^_*P*_ = 0.008), indicating that overall males and females had similar total IDT scores. There was a significant group by sex/gender interaction (*F*(1, 78) = 5.41, *p* = 0.023, *η*^*2*^_*P*_ = 0.065).

Planned contrasts showed that neurotypical boys had significantly higher total IDT scores than boys with autism/high autistic traits (*t*(42) = 3.19, *p* = 0.003, with a very large effects size, *d* = 0.96). There was no significant difference between neurotypical girls and girls with autism/high autistic traits (*t*(37) = − 0.17, *p* = 0.870), and with a negligible effect size, *d* = 0.05). In addition, girls with autism/high autistic traits had significantly higher total IDT scores than boys with autism/high autistic traits (*t*(38) = 2.20, *p* = 0.035, *d* = 0.71), despite having very similar levels of parent-reported autistic traits (*t*(37) = − 0.26, *p* = 0.797, *d* = 0.08). There was no significant difference between neurotypical girls and boys (*t*(41) = − 1.08, *p* = 0.284, *d* = 0.33). The results were the same when the covariate was not included in the analysis.

### Discrepancy Method (‘Compensatory Camouflaging’)

A 2X2 between-subjects ANCOVA was used to explore group and sex/gender differences in social cognition using RMET-C scores, with the factors of 2 Sex/Gender (girls; boys) and 2 Group (neurotypical; autism/high autistic traits), and the covariate of full-scale IQ. There was no significant main effect of group (*F* = 0.19, *p* = 0.662) or sex/gender (*F* = 0.13, *p* = 725), and no interaction between the two factors (*F* = 0.06, *p* = 0.802). The results were the same when the covariate was not included in the analysis. Therefore, girls with autism/high autistic traits had higher levels of social reciprocity than boys with autism/high autistic traits, despite having similar levels of social-cognitive ability (theory of mind).

To test whether there was differences in IQ between participants who engaged in high and low levels of compensatory camouflaging, groups were split at the mean for reciprocity (IDT scores = 2.78) and theory of mind (RMET scores = 18.11). An independent samples t-test was run to compare those with low (i.e., below the mean) theory of mind and reciprocity (low compensatory camouflaging group) to those with low theory of mind and high (i.e., above the mean) reciprocity (high compensatory camouflaging group). Table 3 shows that participants with autism/high autistic traits who engaged in high levels of compensatory camouflaging had higher IQ scores than participants with autism/high autistic traits who engaged in low levels of compensatory camouflaging. This comparison, although not significant (*t*(21) = 1.53, *p* = 0.142), represented a medium-to-large effect (*d* = 0.64). In contrast, neurotypical participants who engaged in high levels of compensatory camouflaging had very similar IQ scores to neurotypical participants who engaged in low levels of compensatory camouflaging. This comparison was also not significant (*t*(19) = 0.19, *p* = 0.849) and represented a very small effect (*d* = 0.08). In addition, the female-to-male ratio was higher in the high compensatory camouflaging group (5 females, 6 males) than the low compensatory camouflaging group (4 females, 8 males).

**Table 3:** IQ scores and gender ratio for children and adolescents in the autism/high autistic traits and neurotypical groups who demonstrated low and high levels of compensatory camouflaging

	Autism/high autistic traits (*n* = 23)		Neurotypical (*n* = 21)	
	Low (*n* = 12)	High (*n* = 11)	Low (*n* = 10)	High (*n* = 11)
Full-scale IQ (*SD*)	87.25 (15.43)	96.73 (14.26)	105.30 (11.43)	106.36 (13.55)
Gender (M:F) ratio	8:4	6:5	4:6	5:6

High compensatory camouflaging is defined as low theory of mind and high reciprocity; low compensatory camouflaging is defined as low theory of mind and reciprocity

### Associations Between Variables

Table 1 shows there were no significant correlations between social reciprocity and full-scale IQ, or participant age. There was also no relationship between social-cognitive ability and age. These results were the same when groups were considered separately. There was a significant positive correlation between full-scale IQ and theory of mind scores (see Table 1). Considering groups separately, this positive association was only significant for participants with autism/high autistic traits (*r* = 0.601, *p* < 0.001). In contrast, there was a negligible non-significant negative correlation for neurotypical participants between IQ and theory of mind (*r* = -0.130, *p* = 0.400).

## Summary of Results

The aim of this study was to investigate sex/gender differences in camouflaging in children and adolescents, using both observational/reflective (behavioural camouflaging) and discrepancy (compensatory camouflaging) methods. The findings provide evidence of camouflaging in girls—but not boys—with autism/high autistic traits, for both operationalisations. Specifically, they highlight that girls with autism/high autistic traits displayed higher levels of social reciprocity than boys with autism/high autistic traits. Additionally, girls with autism/high autistic traits showed more similar levels of social reciprocity to neurotypical girls than males with autism/high autistic traits relative to neurotypical males, despite having very similar levels of parent-reported autistic traits (behavioural camouflaging). Further, girls with autism/high autistic traits had similar levels of social cognitive ability (theory of mind) to boys with autism/high autistic traits, despite increased reciprocal social behaviour (compensatory camouflaging). The results also suggested that, irrespective of sex/gender, children and adolescents with autism/high autistic traits who demonstrated compensatory camouflaging (i.e., low theory of mind and high social reciprocity) had higher IQs compared with individuals who demonstrated low levels of compensatory camouflaging (i.e., low theory of mind, low social reciprocity). However, this latter finding was non-significant, despite group differences reflecting a medium-to-large effect.

## Discussion

The current findings replicate a growing body of literature that suggests camouflaging is a key part of the female autism phenotype and is more prevalent in autistic females than males (e.g., Hull et al. 2019a, 2020 for a review; Lai et al. 2016). The results also fit with an emerging set of studies that have demonstrated greater levels of compensatory camouflaging in autistic females (e.g., Lai et al. 2016), and with the proposition of an ‘improved behavioural presentation of a neurodevelopmental disorder despite persisting core deficit(s) at cognitive and/or neurobiological levels’ (Livingston and Happé 2017, p. 731). This study extends previous findings by investigating camouflaging in children and young adolescents with high levels of autistic traits, without a clinical diagnosis. We argue that children and adolescents who are camouflaging are less likely to have received a diagnosis, because they would have, to some degree, masked their social difficulties.

One previous study has investigated sex/gender differences in social reciprocity in autism (van Ommeren et al. 2017). Their results were consistent with evidence for camouflaging in girls with autism and were similar to those reported in the current paper, highlighting that autistic girls showed greater social reciprocity than autistic boys, despite having similar levels of parent or teacher reported autistic traits. In addition, they also showed that the difference in reciprocity between girls with and without autism was smaller compared to boys with and without autism. However, our results found no significant social reciprocity differences between girls with and without autism/high autistic traits. This difference may be partly explained by girls with autism/high autistic traits in this study having slightly higher reciprocity scores than those in the van Ommeren et al. (2017) study, which may reflect that all participants attended mainstream schools, and some did not have a formal diagnosis of autism. In contrast, girls in the van Ommeren et al. (2017) study all had clinical diagnoses and attended special schools. However, there were other key differences between the studies. For example, neurotypical girls in our study had lower reciprocity scores than those in the van Ommeren paper, which may reflect developmental differences, as the girls in this study were (on average) 4 years younger.

Lai et al. (2016) found that autistic adult males and females had similar theory of mind scores, but females had significantly better behavioural presentation (as demonstrated by significantly lower ADOS social communication scores). Our findings replicate these in a sample of children and adolescents, with a different behavioural measure. Livingston et al. (2018) also found that autistic adolescents who demonstrated higher levels of compensatory camouflaging had significantly higher IQ scores than those who demonstrated low levels of compensatory camouflaging, and that females were more likely to be in the high (than low) compensatory camouflaging group (although this latter finding did not reach statistical significance). Within our sample, children and adolescents with autism/high autistic traits who engaged in high levels of compensatory camouflaging also had non-significantly higher IQ than those who engaged in low levels of compensatory camouflaging. In addition, we found that females with autism/high autistic traits were more likely to be in the high, rather than low, compensatory camouflaging group. However, it must be noted that this IQ difference is based on a small number of participants, and therefore should be interpreted with caution.

In the current study, consistent with previous research, IQ scores were positively correlated with theory of mind scores in the autism/high autistic traits group (reviews by Baker et al. 2014; Hadwin and Kovshoff 2015). This association was not evident in the neurotypical group. This difference between groups raises the possibility that individuals with autism/high autistic traits who performed well in the theory of mind task were more able to compensate for core theory of mind difficulties (see Swettenham 2000 for a similar argument). For example, it is possible that individuals with good memory (a component of IQ) may be able to remember many facial expressions, as well as which emotions they are associated with, without fully or intuitively understanding the emotion or expression. In support, several studies have shown that individuals with autism who can pass theory of mind tasks do not utilise these skills automatically (Senju et al. 2009) or in daily life (e.g., Plumet and Tardiff 2005).

## Implication for Practice

One important implication of these findings is that girls with social communication difficulties may be missed by practitioners, particularly when only relying on observations of behaviour, which may limit the opportunities for them to receive needs-driven support. Indeed, several studies have indicated that girls tend to be diagnosed later than autistic boys (Begeer et al. 2013; Dworzynski et al. 2012; Russell et al. 2011). These results suggest that practitioners should include measures that extend beyond the behavioural domain when assessing children with potential social communication difficulties, such as measures of social cognition.

We hope these findings will raise awareness of the camouflaging phenomenon, so that practitioners are able to identify individuals who may have social communication difficulties earlier in development, put appropriate support in place, and facilitate the best possible developmental outcomes. Early identification of autism is important because both late identification and camouflaging have been linked with mental health difficulties including in autistic adults (Hull et al. 2017, 2019b), late-diagnosed females (Bargiela et al. 2016) and autistic adolescents (Cridland et al. 2014; Tierney et al. 2016). Conversely, camouflaging has also been found to facilitate positive outcomes, such as success in jobs and relationships (e.g., Livingston et al. 2019). It is therefore important for professionals who work with autistic individuals and their families to elicit the views and aspirations of individuals themselves with respect to the potential costs associated with camouflaging behaviours, as well as whether these behaviours are helping them to achieve personally important goals.

A further implication is highlighted for stakeholders in education and mental and health services, where professionals such as Educational Psychologists could include relevant sex/gender difference research in trainings for teaching staff in school. This dissemination is particularly important as school staff will often be the first to notice difficulties in children before referring to other professional services. A final point relates to the ‘double-empathy problem’, which suggests that autistic and non-autistic individuals may have difficulty interacting with each other due to their differing world-views (Milton et al. 2018). One way this gap can be bridged is through non-autistic individuals attempting to understand the experiences and worldviews of autistic individuals. A greater understanding of the differences that autistic individuals may display could lead to greater societal acceptance of neurodiversity, such that autistic individuals feel less need to camouflage.

## Limitations and Future Research

In the current study, participants with autism/high autistic traits had significantly lower verbal IQ than neurotypical participants. Therefore, verbal IQ was included as a covariate in the analyses, and a significant group by sex/gender interaction was found. The sample size was also modest, meaning it was underpowered to detect differences in IQ between those participants with autism/high autistic traits that engaged in high, versus low, levels of compensation. However, based upon a power analysis, the a priori target number of participants was met that allowed us to meet the primary aim of detecting a significant interaction effect between group and sex/gender.

Another limitation is that theory of mind scores were not significantly lower in the autism/high autistic traits than neurotypical group. This result might suggest that the task was not sensitive to theory of mind differences or, alternatively, both groups simply had similar theory of mind abilities. A previous (unpublished) study found that the RMET-C was only able to differentiate between neurotypical and at-risk children when employing an open-ended format (Cassels 2015), as opposed to forced-choice format, as used in this study. Future research should employ the RMET-C using an open-ended format, a wider range of theory of mind tasks—including more complex ones—as well as other social-cognitive tasks, to explore whether group or sex/gender differences are found. It is possible that completing theory of mind tasks is more effortful for autistic individuals, and therefore it would be useful to include measures of effort (e.g., time taken to complete task) in future research.

A strength of this study is that we included participants who had autistic traits who had not received a diagnosis. However, the majority had come to clinical attention (as most were under assessment for ASD). Arguably, those engaging in camouflaging most successfully will not have come to clinical attention at all. As such, it would be useful for future research to include children with high autistic traits who were not known to clinical services. In addition, future research should also include a sufficient number of participants to compare males and females with diagnoses to those with high autistic traits without a diagnosis, as well as neurotypical males and females.

It should also be noted that the IDT only achieved moderate-to-good test–retest reliability in the validation study (van Ommeren et al. 2015), which raises questions about whether this measure provides a reliable and stable assessment of overall social reciprocity. However, the IDT is an interactive test, designed to measure spontaneous behaviour, and therefore slightly different responses may be expected on different administrations. Moreover, to our knowledge, the IDT is the only tool validated to directly and objectively measure social reciprocity in autistic children and so was considered more appropriate than alternative methods of measuring social reciprocity (e.g., questionnaires). Another limitation of the current study is that our inter-rater reliability checks were not evenly distributed across groups. Despite this, our inter-rater checks yielded an excellent overall reliability coefficient, which was also found in the validation study of the IDT (van Ommeren et al. 2015).

A suggestion for future research, is to ask autistic children to complete the IDT with another child to maximise ecological validity, as well as ensure inter-rater reliability checks are evenly distributed across all groups. For example, it is possible that children may have a different behavioural presentation when interacting with peers, rather than an unfamiliar adult researcher. It would also be useful to directly compare behavioural tasks (such as the IDT), with observation-based measures, such as the Playground Observation of Peer Engagement (POPE; see Dean et al. 2017), as well as self-report measures of social camouflaging, such as the Camouflaging Autistic Traits Questionnaire (CAT-Q, Hull et al. 2019a, b). This would help ascertain the extent to which behavioural tasks correspond to real-life social interaction and how successful individuals are in their intentions to camouflage. Finally, it is also important for future research to attempt to disentangle the effects of sex and gender on camouflaging, for example, by including participants who do and do not identify their gender with their biological sex.

## Conclusion

In conclusion, this study provides further evidence of greater camouflaging in females with autism. To the researchers’ knowledge, this is only the second study to investigate camouflaging that has included child and adolescent participants with high autistic traits, without diagnoses (see also Livingston et al. 2018). These findings may partly explain why many autistic females are diagnosed at a later age than their male counterparts, limiting their opportunities to receive support.

## Footnotes

1 The American Psychological Association (2011) note that sex refers to an individual’s biological status as male or female’, whereas gender refers to the “socially constructed roles, behaviours, activities, and attributes that a given society considers appropriate for boys and men or girls and women” (p. 1). In the current paper the term ‘sex/gender’ is used throughout to acknowledge the overlap between the two terms in autism research (Lai et al. 2015; Springer et al. 2012).

2 Previous research suggest that there is no one preferred way of describing autism within the broader autism community, therefore both person-first (e.g., individual with autism) and identity-first (e.g., autistic female) will be used in this paper (Kenny et al. 2016; Vivanti 2019).
